# Supplementary material for: The Effect of Mice Adaptation Process on the Pathogenicity of Influenza A/South Africa/3626/2013 (H1N1)pdm09 Model Strain
Source: Int J Mol Sci. 2023 Dec 12;24(24):17386. doi: 10.3390/ijms242417386 (PMC10743444; doi:10.3390/ijms242417386)
Supplement: Supplementary file 1 [file ijms-24-17386-s001.zip › ijms-2724696-supplementary.pdf]

International Journal of Molecular Sciences

MDPI

Table S1. Primers used for full-genome sequencing of A/South Africa/3626/2013 virus genome.

Gene	Fragment	Primers for RT-PCR	Sequence 5′→3′	Primers for reaction with Big Dye kit	Sequence 5′→3′
PB2	PB2-1	PB2 Uni F1	GATCGCTCTTCAGGGAGCGAAGCAGGTC [Hoffmann DOI: 10.1007/s007050170002]	PB2 Uni F1	GATC GCTCTTCAGGGAGCGAAAGCAGGTC [Hoffmann DOI: 10.1007/s007050170002]
		PB2 R1322	TGCATGGGGTTCAGTCTCTG	PB2 F759	CACTCCAGGAGGAGAAGTGAG
				PB2 R1140	TGTTGCTCTTCTCCCAACCATTGT
	PB2-2	PB2 F1063	ACGGGCAATCTCCAAACA	PB2 F1063	ACGGGCAATCTCCAAACA
		PB2 Uni R	ACTG GCTCTTCTATT AGTAGAAACAAGG TCGTTT [Hoffmann DOI: 10.1007/s007050170002]	PB2 R1477	CCATCTTGCTGACTCTTA
				PB2 Uni R	ACTG GCTCTTCTATT AGTAGAAACAAGG TCGTTT [Hoffmann DOI: 10.1007/s007050170002]
PB1	PB1-1	PB1 F1	GAGCGAAAGCAGGCAAACCA	PB1 R431	TTTCTGTTTAATGTCCAATCA
		PB1 R1071	TGC CAT TTT RTT TGA GAA CAT TAT [Matyushenko et al doi.org/10.1371/journal.pone.0180497]	PB1 F175	GAAAAGGGGAAGTGGACAACAA
				PB1 R835	GCCCAGACTGTTCAAG
	PB1-2	PB1 F594	AGT AAG RGA CAA CAT GAC CAA GAA [[Matyushenko et al doi.org/10.1371/journal.pone.0180497]	PB1 F594	AGT AAG RGA CAA CAT GAC CAA GAA [[Matyushenko et al doi.org/10.1371/journal.pone.0180497]
		PB1 Uni R	ACTG GCTCTTCTATT AGTAGAAACAAGG CATTTT [Hoffmann DOI: 10.1007/s007050170002]	PB1 R1536	ATTAGCCACAAATCCATAGCG
				PB1 R1071	TGC CAT TTT RTT TGA GAA CAT TAT [Matyushenko et al doi.org/10.1371/journal.pone.0180497]

				PB1 Uni R	ACTG GCTCTTCTATT AGTAGAAACAAGG CATTTT [Hoffmann DOI: 10.1007/s007050170002]
				PB1 R2273	ATGGTGGAACAGATCTTCATGATCTC [Deng 2011 DOI: 10.1371/journal.pone.0023400]
PA	PA-1	PA Uni F1	GATCGCTCTTCAGGGAGCGAAAGCAGGTAC [Hoffmann DOI: 10.1007/s007050170002]	PA F62	TCGTCGAGCTTGCGGAAAA
		PA R1709	AACATGGGCCTCGACACTTG	PA F593	CCTTTCGTCAGTCCGAAAGAGG [Deng 2011 DOI: 10.1371/journal.pone.0023400]
				PA R619	CTT CGC CTC TTT CGG ACT GAC G [Matyushenko et al doi.org/10.1371/journal.pone.0180497]
				PA R1709	AACATGGGCCTCGACACTTG
	PA-2	PA F1623	GCCACACAAATGGGAAAAATAC	PA F1270	GAATTGACTGATTCAAGCTGG
		PA Uni R	ACTG GCTCTTCTATTAG-TAGAAACAAGGTACTT [Hoffmann DOI: 10.1007/s007050170002]	PA F1623	GCCACACAAATGGGAAAAATAC
				PA F2002	GCTGAATCAAGAAAATTGC
				PA Uni R	ACTG GCTCTTCTATTAG-TAGAAACAAGGTACTT [Hoffmann DOI: 10.1007/s007050170002]
HA	HA-1	HA Uni F1	GATCGCTCTTCAGGGAGCAAAAGCAGGGG [Hoffmann DOI: 10.1007/s007050170002]	HA Uni F1	GATCGCTCTTCAGGGAGCAAAAGCAGGGG [Hoffmann DOI: 10.1007/s007050170002]
		HA R903	CGTGGACTGGTGTATCTGAAATGA	HA F347	AACGTGTTACCCAGGAGATTTCA
				HA R438	TGGGGAATATCTCAAACCTT
				HA R903	CGTGGACTGGTGTATCTGAAATGA
		HA F776	GACACTAGTAGAGCCGGGAGACAA	HA Uni R	ACTGGCTCTTCTATT AGTAGAAACAAGG GTGTTTT [Hoffmann DOI: 10.1007/s007050170002]
		HA Uni R	ACTGGCTCTTCTATT AGTAGAAACAAGG GTGTTTT [Hoffmann DOI: 10.1007/s007050170002]	HA F776	GACACTAGTAGAGCCGGGAGACAA
				HA F1312	AAGTTGATGATGGATTCCTG

				HA R1462	ATTTCCTTGGCATTGTTTT
NP	NP-1	NP F1	GGAGCAAAAGCAGGGTAGATAATC	NP F1	GGAGCAAAAGCAGGGTAGATAATC
		NP R1195	TGCTTCTTAGTTCCAGGGTAT	NP R789	TGGGTTTCGACTTTCTCTTACTTG
				NP R1195	TGCTTCTTAGTTCCAGGGTAT
	NP-2	NP F1020	GAGTCAATTGGTATGGATGGC	NP F1091	GAAAGAAAGTGATCCCAAGAG
		NP Uni R	ACTGGCTCTTCTATT AGTAGAAACAAGG GTATTTT [Hoffmann DOI: 10.1007/s007050170002]	NP Uni R	ACTGGCTCTTCTATTAGTAGAAACAAGGG-TATTTT [Hoffmann DOI: 10.1007/s007050170002]
NA	NA-1	NA Uni F1	GATCGCTCTTCAGGG AGCAAAAGCAGG AGT [Hoffmann DOI: 10.1007/s007050170002]	NA F290	TCTCTGCCCTGTTAGTGGATG
		NA R880	TCACTAGAATCAGGATAACAGGA	NA R377	TTCCCTTATGACAAACACA
				NA R880	TCACTAGAATCAGGATAACAGGA
	NA-2	NA F517	TTCCCTCTCCATACAACTCAA	NA F517	TTCCCTCTCCATACAACTCAA
		NA Uni R	ACTG GCTCTTCTATT AGTAGAAACAAGGAG-TTTTTT [Hoffmann DOI: 10.1007/s007050170002]	NA F1020	AGTTGTGGTCCAGTATCGTCTAAT
				NA R1219	GACCACTCATTTATCCCTACGA
M		M Uni F1	GAT CGC TCT TCA GGG AGC AAA AGC AGG TAG [Hoffmann DOI: 10.1007/s007050170002]	M F260	TTTATCCAAAATGCCCTAAATG
		M Uni R	ACTGGCTCTTCTATTAGTAG AAACAAGGTAGTTTTT [Hoffmann DOI: 10.1007/s007050170002]	M R702	CTGGAGCTAGGATGAGTCCCA
NS		NS Uni F1	GATCGCTCTTCAGGGAGCAAAAGCAGGGTG [Hoffmann DOI: 10.1007/s007050170002]	NS R559	ATTGCATTTTTGACATCCT [Matyushenko et al doi.org/10.1371/jour-nal.pone.0180497]
		NS Uni R	ACTGGCTCTTCTATTAG-TAGAAACAAGGGTGTTTTT [Hoffmann DOI: 10.1007/s007050170002]	NS F108	GGTGATGCCCCATTCCTTG

Table S2: Raw data of the two–way hemagglutination inhibition test performed with serum samples obtained from mice infected with WT or MA variants of SA virus in different doses.

**HAI test with 4 HAU of SA-WT as antigen**												
Infection dose, $\log_{10}$ EID_{50}/mL	**HAI titer of each serum obtained on the 21st day, from mice (m) infected with SA-M5**										Average	SD
	m1	m2	m3	m4	m5	m6	m7	m8	m9	m10		
4.4	1280	320	640	640	160						608	429.3251
3.4	640	640	160	160	160	320	320	640	160		355.5556	223.1093
2.4	160	40	320	80	320	320	80	160	40	640	216	187.806
Infection dose, $\log_{10}$ EID_{50}/mL	**HAI titer of each serum obtained on the 21st day, from mice (m) infected with SA-WT**										Average	SD
	m1	m2	m3	m4	m5	m6	m7	m8	m9	m10		
4.4	160	320	160	160	80	640	80	160	160		213.3333	174.356
3.4	40	160	320	80	80	160	80	80	320	320	164	113.842
2.4	10	5	20	10	80						25	31.22499
**HAI test with 4 HAU of SA-M5 as antigen**												
Infection dose, $\log_{10}$ EID_{50}/mL	**HAI titer of each serum obtained on the 21st day, from mice (m) infected with SA-M5**										Average	SD
	m1	m2	m3	m4	m5	m6	m7	m8	m9	m10		
4.4	1280	320	640	640	160						608	429.3251
3.4	640	640	160	160	160	320	320	640	160		355.5556	223.1093
2.4	160	40	320	80	320	320	80	160	40	640	216	187.806
Infection dose, $\log_{10}$ EID_{50}/mL	**HAI titer of each serum obtained on the 21st day, from mice (m) infected with SA-WT**										Average	SD
	m1	m2	m3	m4	m5	m6	m7	m8	m9	m10		
4.4	160	320	160	160	80	640	80	160	160		213.3333	174.356
-3	40	160	320	80	80	160	80	80	320	320	164	113.842
-4	10	5	20	10	80						25	31.22499

Groups of 10 mice were infected with 3 different doses of two tested viruses and then lethality was monitored for 14 days. The surviving animals were used for serological study: the blood was collected from surviving animals on day 21 post-infection. In Table S1, the raw results of the two–way HAI test for each survived mouse are presented; the HAI values are indicated for each serum, for both antigens. There were no differences in antigenic properties detected for the two viruses: the HAI titers were the same for each serum regardless of the antigen used. Significant differences in the immunogenicity of the two viruses were detected; sera of animals infected with the SA–M5 virus had higher titers with both antigens (described in paragraph 3.5 of the Results section.
